# Functionalization of Titanium with Chitosan via Silanation: Evaluation of Biological and Mechanical Performances

**DOI:** 10.1371/journal.pone.0039367

**Published:** 2012-07-31

**Authors:** Pauline Renoud, Bérangère Toury, Stéphane Benayoun, Ghania Attik, Brigitte Grosgogeat

**Affiliations:** 1 Laboratoire des Multimatériaux et Interfaces, Université Lyon 1, Université de Lyon, Villeurbanne, France; 2 Laboratoire de Tribologie et Dynamique des Systèmes, Ecole Centrale de Lyon, Ecully, France; 3 Service de Traitements et de Consultations Dentaires, Hospices Civils de Lyon, Lyon, France; Université de Technologie de Compiègne, France

## Abstract

Complications in dentistry and orthopaedic surgery are mainly induced by peri-implant bacterial infections and current implant devices do not prevent such infections. The coating of antibacterial molecules such as chitosan on its surface would give the implant bioactive properties. The major challenge of this type of coating is the attachment of chitosan to a metal substrate. In this study, we propose to investigate the functionalization of titanium with chitosan via a silanation. Firstly, the surface chemistry and mechanical properties of such coating were evaluated. We also verified if the coated chitosan retained its biocompatibility with the peri-implant cells, as well as its antibacterial properties. FTIR and Tof-SIMS analyses confirmed the presence of chitosan on the titanium surface. This coating showed great scratch resistance and was strongly adhesive to the substrate. These mechanical properties were consistent with an implantology application. The Chitosan-coated surfaces showed strong inhibition of *Actinomyces naeslundii* growth; they nonetheless showed a non significant inhibition against *Porphyromonas gingivalis* after 32 hours in liquid media. The chitosan-coating also demonstrated good biocompatibility to NIH3T3 fibroblasts. Thus this method of covalent coating provides a biocompatible material with improved bioactive properties. These results proved that covalent coating of chitosan has significant potential in biomedical device implantation.

## Introduction

Bacterial adhesion on medical devices and implants is a main source of complications in dentistry and orthopaedic surgery [1], [2]. For example, reports on loss of dental implants clearly define peri-implantitis as a major cause of the implant failure [1], [3]. Peri-implantitis is defined as an inflammatory reaction with the loss of supporting bone in the tissues surrounding a functioning implant [4]. The risk of peri-implantitis is increased with patients showing complicating factors such as diabetics [5] or smokers [6]. There is currently no preventive treatment against peri-implantitis, but only curative treatment (treatment with antibiotics or antiseptics). Titanium and its alloys are typically used for implants because of their superior biocompatibility, first-rate corrosion resistance, and good mechanical properties [7], [8]. However, titanium implants do not prevent peri-implant infections [9]. Therefore, an alternative is needed using titanium implants with a bioactive coating that would have antiseptic properties. Implants should also allow for strong adhesion of peri-implant soft tissues which helps to prevent bacterial colonization and subsequent chronic inflammation [10]. That is why the implant should also allow for improved gingival cell adhesion on its surface. The use of natural polymers seems to be an attractive option because of their good biocompatibility.

Chitosan is a biological, biodegradable and nontoxic polymer [11], [12]. It is already used in animal medicine as a healing agent or in cosmetics. Two other advantages are that it is inexpensive and abundant. It is obtained from the deacetylation of chitin, a polymer in the exoskeletons of crustaceans [13]. It has been shown that chitosan can increase the growth and attachment of gingival cells [14], [15]. Plus, the positive charge of the amino groups along the biopolymer chain is reported to confer unique antibacterial properties [16]. It is therefore a perfect candidate for coated implants.

The major challenge of this type of coating is the attachment of chitosan to a metal substrate. Studies have been conducted on chitosan simply deposited on the metal surface with a weak bond (0.5 MPa) [17]. Chitosan films can also be formed by coating the substrate with a silane molecule. 3-Aminopropyltriethoxysilane (APTES) is one silane molecule commonly used in the biomedical literature to bond an assortment of materials [18]–[20]. APTES then reacts with a linker molecule, the glutaraldehyde, which will form a covalent bond with the chitosan. Using a silanation significantly increases the strength of the bond between chitosan and the metal substrate (1.5–1.8 MPa) [17]. There is however another method using a silane with one step less. This method involves the grafting of triethoxysylilbutyraldehyde (TESBA) which is directly linked with chitosan [21].

The purpose of this study was to achieve the coating of chitosan using TESBA as silane and to control the chemical composition of the coated surface. To investigate this coating any further it was important to check its adhesion to the metal substrate and its mechanical properties. Then the biological properties of coated-chitosan material had to be demonstrated. There is no evidence that the coating does not affect these chitosan properties [22]. As soft tissues are mainly formed by fibroblasts, the biocompatibility of the implant and these cells must be tested. The antibacterial properties should also be assessed on oral bacteria. Generally, the developing microbiota around implants closely resembles the microflora of naturally remaining teeth [23]. But some germs seem to be principally involved in the development of peri-implantitis [24]. If the biological properties against these bacteria prove to be increased compared to a pure titanium substrate, the covalent coating would be a major improvement and would pave the way for a new type of implant material.

**Figure 1:**
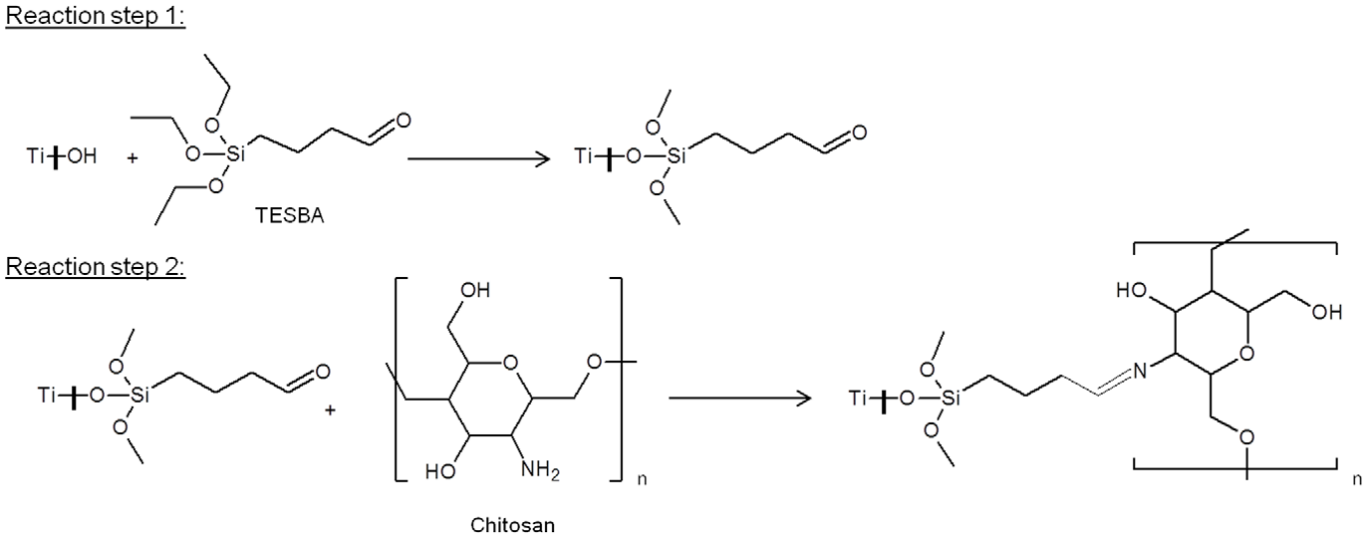
Reaction scheme allowing the covalent bonding of chitosan to titanium surface.

**Figure 2:**
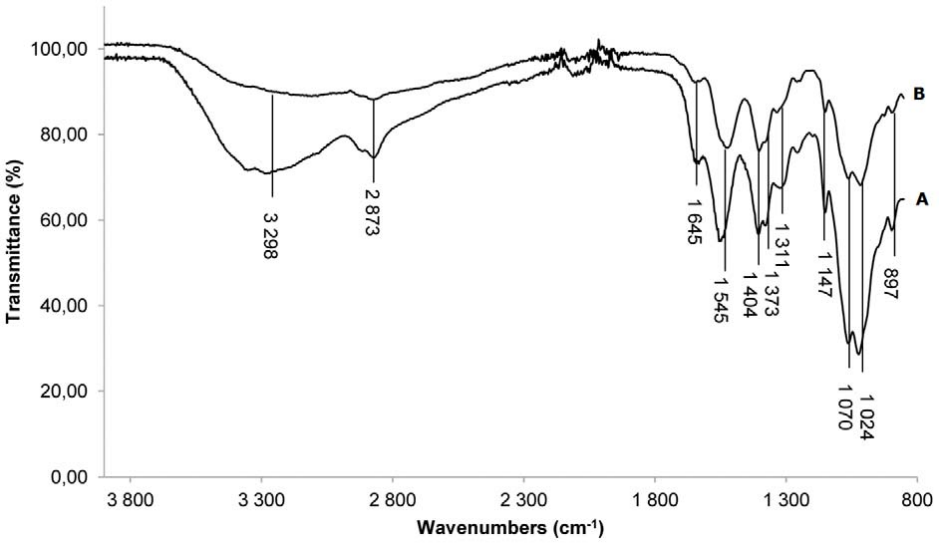
FTIR-ATR spectra. (A) chitosan. (B) chitosan–coated sample.

## Materials and Methods

### 1. Materials

Titanium foils were purchased from Tekka (France). Toluene, ethanol, low molecular weight chitosan, DMSO, HDMS, Glutaraldehyde, Sodium Cacodylate, resazurin and propidium iodide were obtained from Sigma-Aldrich while TESBA were purchased from ABCR (Germany). Media, buffer solutions, Trypsine/EDTA, Triton X100 and antibiotics were purchased from PAA Company (Austria). Staining solutions were obtained from In vitrogen (USA). Bacteria were obtained from Institut Pasteur (France) and fibroblasts cells were purchased from American Type Culture Collection (ATCC, USA).

### 2. Surface functionalization of titanium

Titanium foils were sonicated for 30 min in a 50∶50 (v/v) acetone/ethanol solution. They were left for 15 min in a 7∶3 (v/v) hydrogen peroxide/sulfuric solution at room temperature (Piranha solution). Then metal coupons were strongly rinsed in ultra-pure water. To attach silane on surfaces, dried titanium piranha treated samples were submerged in a 2% (v/v) solution of TESBA in extra-dry toluene and allowed to react for 24 h. Following the 24 h reaction time, the coupons were placed in pure anhydrous toluene and sonicated for 30 min. The procedure of sonication was repeated twice more using fresh anhydrous toluene, for a total sonication time of 90 min. To remove any residual toluene, the metal coupons were rinsed with ethanol and then dried 10 minutes at room temperature. Then, the substrates were dipped one time in a solution of 4 wt.% chitosan, 2 wt.% acetic acid, and 94 wt.% deionized water through a dip-coater (v = 3 mm/s). The chitosan-coated samples were then allowed to dry at 80°C for 4 hours.

**Table 1:** Proportion of chitosan specific fragments measured by Tof-SIMS.

Fragments	Chitosan solution	Chitosan coated sample
C_2_H_5_O_2_	18,38±1,58%	14,27±0,04%
C_5_H_6_NO	33,99±0,45%	31,76±0,04%
C_5_H_5_O_2_	14,54±0,26%	19,02±0,13%
C_4_H_6_NO_2_	6,98±0,04%	7,58±0,04%
C_5_H_6_NO_2_	8,31±0,11%	12,87±0,05%
C_6_H_10_NO_3_	17,80±1,33%	14,50±0,14%

**Figure 3:**
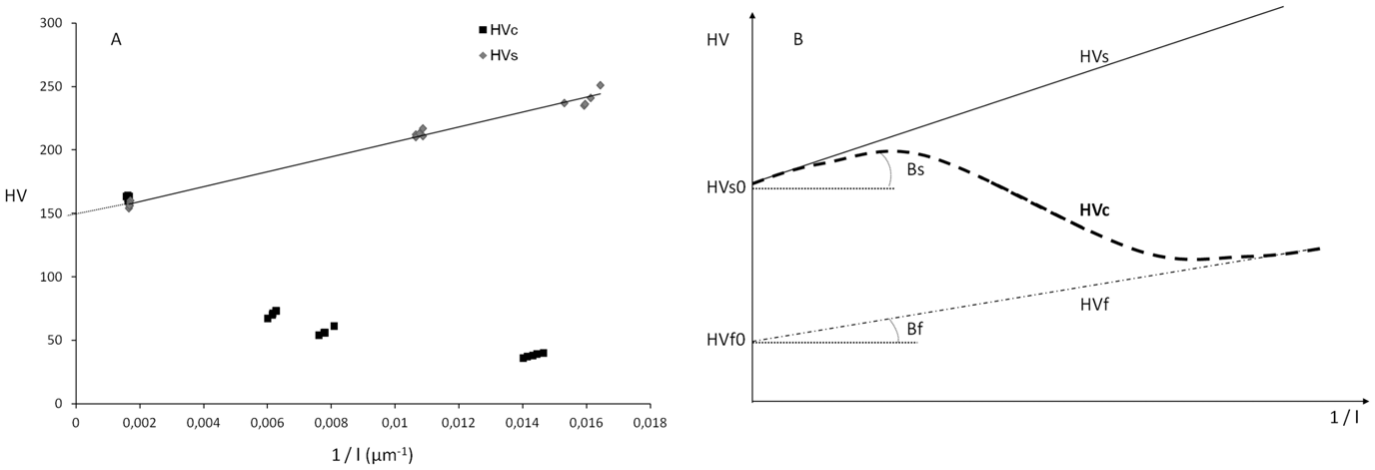
Graphs displaying the evolution of the Vickers Hardness (HV) versus inverse of the diagonal of the indent (1/l). (A) Measurements on the composite (HVc) and on the substrate (HVs). (B) Scheme of the theoretical curves. HVs represents the curve of a hard substrate and HVf the curve of a soft film. HVc is the curve of the composite film-substrate: for high load (l → ∞), HVc ∼ HVs, for very low load (l → 0), HVc ∼ HVf.

### 3. Surface chemistry characterization

#### 3.1 FTIR-ATR

The surface functional group characterization of the coated samples was investigated by Fourier transform infrared (FTIR) spectroscopy-Attenuated Total Reflectance (ATR). FTIR-ATR measurements were recorded on an FTIR 300E (Jasco, France) device at wave numbers ranging from 4000 to 600 cm^−1^. Each FTIR spectrum represented an average of 64 scans. Spectra were recorded on the coated samples and were compared to the spectra obtained from pure chitosan film and pure TESBA solution.

#### 3.2 Tof-SIMS

The presence and homogeneity of TESBA and then of chitosan coatings on titanium were also assessed by means of a Tof-SIMS V instrument (ION-TOF GmbH, Germany). For this study, a pulsed primary ion source of Bi (3) was operated at 25 kV with a pressure of 5.10^−9^ Torr. The primary ion dose density was 3.10^8^ ions/cm^2^. The scanning area of secondary ions was 100 µm×100 µm. Positive secondary ions were detached from the outmost layer of the coating and their respective masses were measured. Pure TESBA and chitosan solution were tested as controls. TESBA solution was sampled under argon atmosphere and TESBA coated samples were washed as in the procedure described in part 2 of Materials and Methods before being tested. The experiments were repeated three times in random locations.

**Figure 4:**
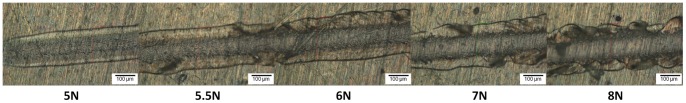
Optical micrographs of scratches observed on chitosan-coated surfaces.

**Figure 5:**
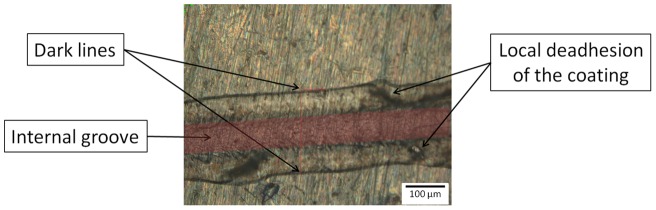
Optical micrograph of scratch observed on chitosan-coated surface. The image is a detail of Figure 4 for a applied load of 5,5 N.

### 4. Mechanical properties characterization

#### 4.1 Profilometry

The film thickness was determined by measuring the interface step between the uncoated and coated samples by profilometry (Dektak, Veeco).

#### 4.2 Vickers Hardness

Conventional Vickers microhardness indentation tests were carried out under loads of 0.1; 0.5; 1 and 30 kg in order to measure titanium surface and coated system hardness. Vickers hardness (HV) value was calculated according to the equation [25],


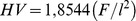
(1)

where F is the applied load (in kgf) and l is the average length of the two diagonals of the recovered cavities (in mm). The corresponding mean contact pressure, H, is defined as ratio of applied force, F and the projected area of contact of the Vickers indenter (l^2^/2). This contact pressure is assumed to be equal to the indentation hardness (more often expressed in MPa) and it was calculated with the following equation [26]:


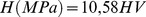
(2)

For each sample, series of 5 indentions for each applied load were made and results expressed as mean value ± standard deviation.

#### 4.3 Scratch test

Scratch resistance of the coatings was assessed using a scratch-tester (Micro Scratch Tester, CSM) with a Rockwell C diamond indenter (cone apex angle 120°, tip radius R = 200 μm) moving along the surface at 2 mm/min and applying a constant force, F. The length of the scratch was 3 mm and the applied force varied between 5 and 8 N. The onset of coating failure was monitored by optical microscopy.

**Figure 6:**
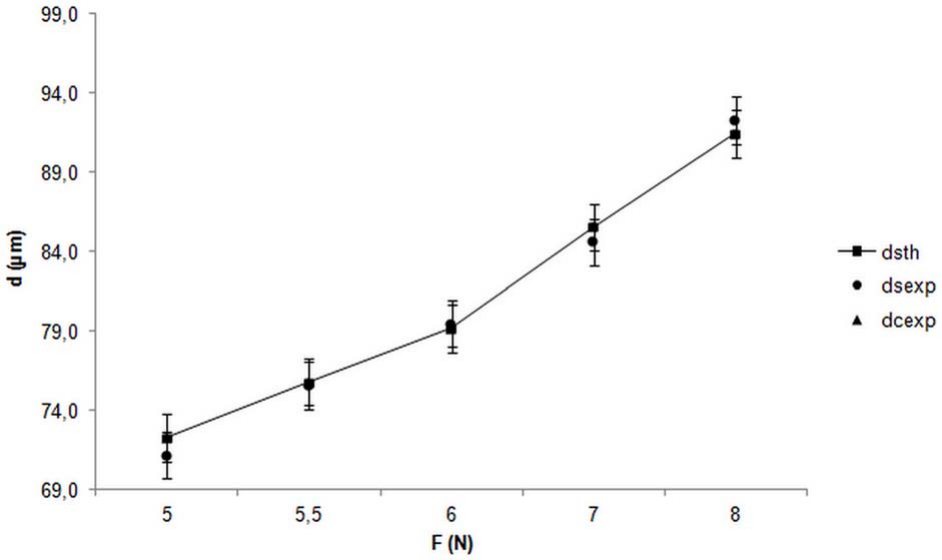
Graph displaying the evolution of the groove diameter (d) versus the applied force (F).

**Figure 7:**
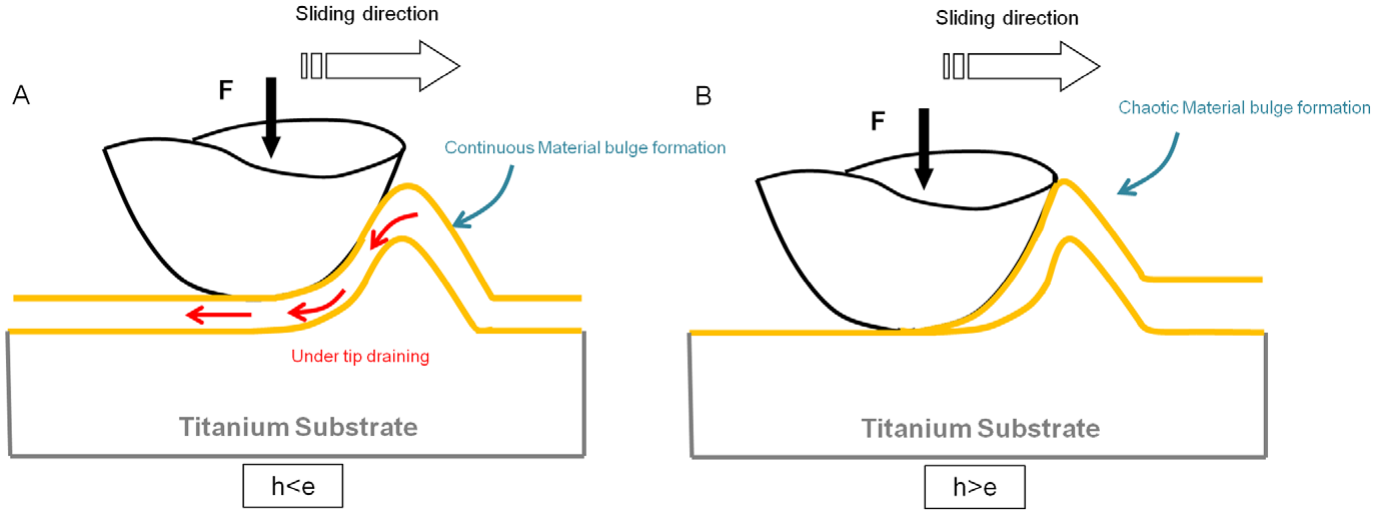
Schemes displaying coating behavior under tip contact with an applied load (F). (A) the burial depth (h) is less than the thickness of the coating (e). (B) the burial depth (h) is greater than the thickness of the coating (e).

### 5. Biological properties evaluation

#### 5.1 Sterilization

For sample sterilization, the samples were sterilized under UV light at 254 nm placed 15 cm from the samples. After 20 min, the samples were turned to another side and then sterilized again for 20 min under UV light.

#### 5.2 Antibacterial Assessment

Antibacterial activity of the samples against *Actinomyces naeslundii* and *Porphyromonas gingivalis* was evaluated by using the optical density method. *P. gingivalis* and *A. naeslundii* were cultured under anaerobic (GENbox anaer, bioMérieux SA, Marcy l'Etoile, France). After 2–3 days for *A. naeslundii* and 6–8 days for *P. gingivalis*, colonies were visible on the plates. The bacterial cells were suspended in water and then placed in a nutrient broth (5 Farland in Schaedler broth). Coated and uncoated samples were placed in 50 ml plastic tubes. 10 ml of bacterial suspension was inoculated into each tube at 37 °C for 32 hours. Samples were taken at specific time intervals to determine the OD at 550 nm in the tubes by using a spectrophotometer Spectronic 20 (Thermo Spectronic, Rochester, New York, USA). OD values being proportional to bacterial growth, the percentage of inhibition was obtained by comparing OD of bacterial suspension in contact with coated and uncoated samples at each time. The experiments were performed 3 times on different days. One-way analysis of variance (ANOVA) was used to assess the normally distributed data and the inhibition percentage are reported as mean ± SD. Statistical significance was accepted at P<0.05.

**Table 2:** Contact depth (h) and diameter (d) for tested loads (F).

F (N)	d (µm)	h (µm)
5	72,3	3,3
5,5	75,8	3,6
6	79,2	3,9
7	85,5	4,6
8	91,4	5,2

**Figure 8:**
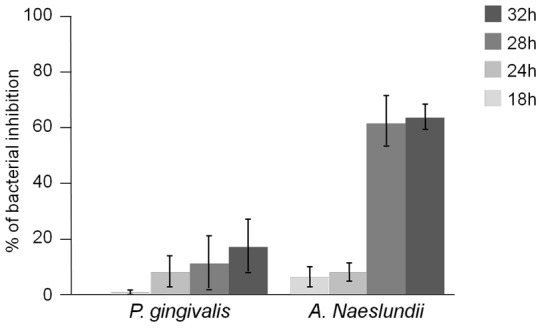
The effects of chitosan coated Ti. on Porphyromonas gingivalis and on Actinomyces naeslundii in liquid medium for 32 hours. Data are expressed in % of bacteria in contact with coated sample compared to the bacteria in contact with control (or percentage of bacterial inhibition). Data represent the means ± SD of three independent experiments. * p<0.01.

#### 5.3 Cell culture

Murine fibroblast cells (NIH3T3) were selected even though recent studies have demonstrated that there are differences between immortalized cells and primary cells in cellular response to the biomaterial surface [27]. However, this cell line has been used in many studies performed on implant devices in order to preliminarily study their biocompatibility [28]–[30]. This type of study could be supplemented by additional studies with primary cells.

Cells were cultured in Dulbecco's modified Eagle medium (DMEM) in the presence of 10% fetal bovine serum (FBS), 2% Penicillin/Streptomycin and 1% Amphotericin B at 37 °C. Cultures were maintained at 37°C under a humidified atmosphere of 5% CO_2_ in air. The medium was changed every 2 days, and the cells were passaged every two days. After reaching confluence, the cells were trypsinized and resuspended in the culture medium. The cells were counted with a Scepter handheld automated cell counter (Millipore, Billerica, USA) and centrifuged at 1200 tr/min for 5 min. After the removal of trypsin, the remaining cell pellets were resuspended in fresh medium for subsequent experiments. One milliliter of cell suspension at a cell density of 1.10^4^ cells/ml was seeded in 12-well microplates containing sterilized materials.

**Figure 9:**
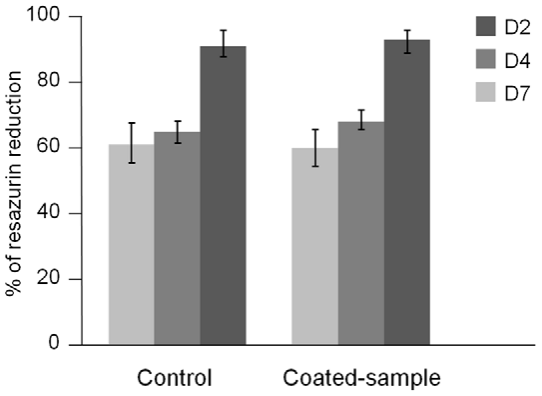
Diagram displaying % of resazurin reduction for uncoated and Chitosan-coated samples. Data are given after 2, 4 and 7 days of NIH3T3 fibroblasts culture. Data are presented as means ± SD.

**Figure 10:**
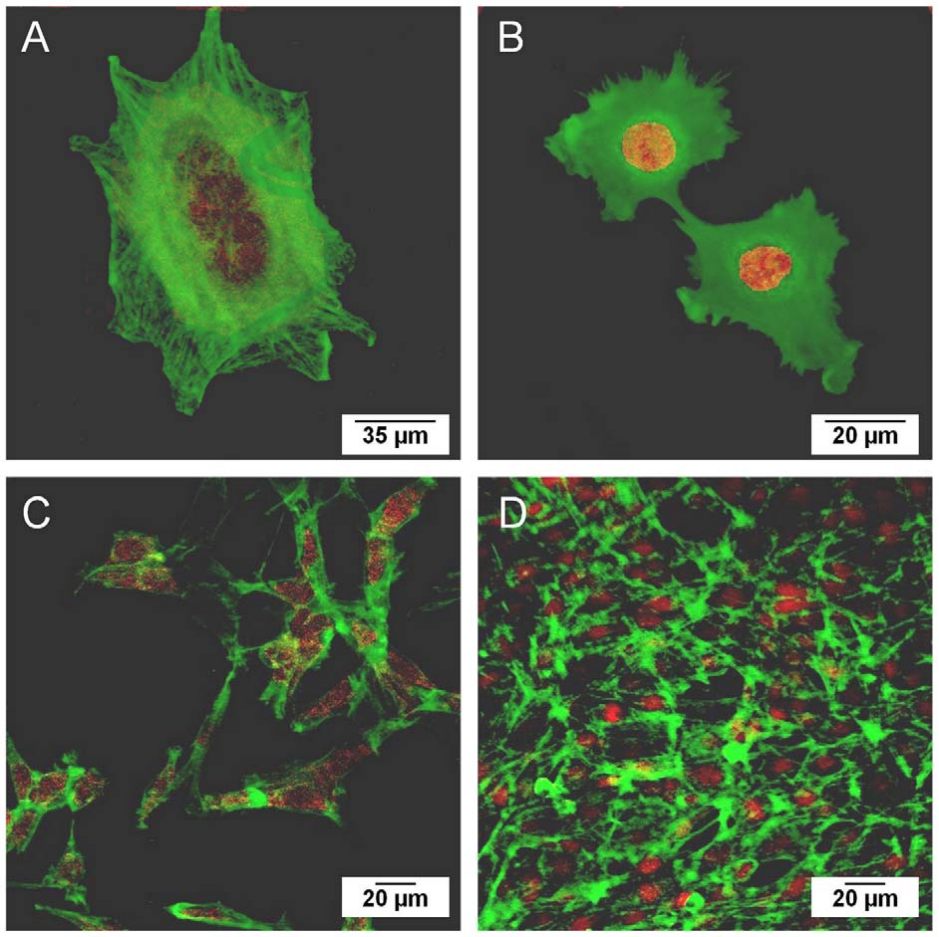
Confocal images of fibroblasts proliferated. (A) 2 days on chitosan-coated surface. (B) 2 days on titanium surface. (C) 4 days on chitosan-coated surface. (D) 7 days on chitosan-coated surface. Actin filaments (green) and nuclei (red) were stained.

#### 5.4 Biocompatibility

#### 5.4.1 Cell proliferation

The resazurin assay is a viability test based on the reduction of the non-fluorescent dye resazurin to a fluorescent dye resorufin by metabolic active cells. One hundred microliters of a filter-sterilized 0.1 mg/ml aqueous solution of resazurin (Sigma) was added to each well. Plates were incubated for 4 hours at 37°C. The absorbance was measured with a Multiskan EX microplate reader (Thermo Scientific, USA) at excitation wavelengths of 570 nm and 630 nm. The results were expressed as a percentage of resazurin reduction. Controls included media plus coated and uncoated substrate, depending on what sample was tested. This percentage was calculated using the previously described formula [31], [32]:


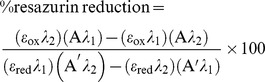


Where, ελ_1_ and ελ_2_ are the molar extinction coefficient of resazurin at 570 and 630 nm, respectively, in the oxidized (εox) and reduced (εred) forms. Aλ_1_ and Aλ_2_ represent absorbance of experimental wells at 570 and 630 nm, respectively. A'λ_1_ and A'λ_2_ represent absorbance of control wells at 570 and 630 nm, respectively. Five samples per time point for each experimental condition were used (n = 5). All experiments were done in duplicate. One-way analysis of variance (ANOVA) and a t-test were used to assess the normally distributed data and the results are reported as mean ± SD. Statistical significance was accepted at P<0.05.

#### 5.4.2 Cell morphology

The substrates were placed into a 12-microwell plate and seeded with fibroblasts at a density of 1.10^4^ cells/ml. The fibroblasts were fixed for confocal laser scanning microscopy observations after 2, 4 and 7 days of culture. The cell-seeded samples were harvested and washed three times with PBS (pH 7.4). The cells were fixed for 10 min by incubating in 3.7% formaldehyde in PBS followed by further washing. The cells were permeabilized with 0.2% Triton X100 in PBS and then blocked with 1% bovine serum albumin in PBS. Actin microfilaments were stained by Alexa Fluor 488 phalloidin (green fluorescence), nuclei were identified by propidium iodide (red fluorescence). Stained cells were visualized with a ZEISS LSM 510 META confocal laser scanning microscope (Carl Zeiss Inc, Germany).

## Results and Discussion

### 1 Surface chemistry characterization

#### 1.1 Control of TESBA grafting

As depicted in Figure 1, the first step of the reaction was TESBA silanation of the titanium surface. FTIR-ATR analysis was used to control the presence of TESBA after this intermediate stage. Unfortunately it was difficult to distinguish the characteristic bands of the silane (1180 cm^−1^ corresponding to Si-CH_2_ stretching vibration mode and 1080 cm^−1^ corresponding to –Si-O-CH_3_ stretching vibration mode) [33] from titanium, which is generally characterized by a broad band (1000–1200 cm^−1^) [34]. The only characteristic peak of the silane that could be observed was the one at 1720 cm^−1^ corresponding to the C  =  O group of the aldehyde. However, the signal of this peak was extremely low (under 90% transmittance). The hypothesis was that a thin monomolecular layer of TESBA was coated to the titanium surface and the coating thickness prevented us from observing a stronger signal.

To gain further insight into the bonding of the TESBA to the titanium surface, ToF-SIMS analysis was done. The TOF-SIMS technique seemed appropriate because it analyses the extreme surface of the sample. Thus, the high sensitivity of this method enables analysis of a very thin coating on the sample surface [35]. TESBA solution provided us with the ion molecular peak (m/z = 234) and two significant fragments (SiC_6_H_15_O_3_, m/z = 163 and SiC_8_H_17_O_3_, m/z = 189). The analysis of the silane-coated surface presented these two fragments. This seemed to verify the presence of TESBA on the surface. In addition, a third significant fragment (SiC_4_H_7_O_4_, m/z = 147) was found on the coated surface which corresponded to the initial silane without any alcoxy-group. Unlike the TESBA solution, which was sampled under argon, the alcoxy-groups on the coating surface were easily hydrolyzed after coating, providing a new fragment. These data verified the presence of TESBA on the sample surface. Considering that the samples were strongly washed, it can be supposed that TESBA was covalently bonded to the titanium substrate.

#### 1.2 Control of chitosan coating

The second step of reaction was the coating of chitosan as presented in Figure 1. The FTIR-ATR spectra of the chitosan-coated surface and pure chitosan film are represented in Fig. 2. The overlapped peaks of N–H stretching and O–H stretching modes that occured around 3300 cm^−1^ in both spectra indicated the presence of hydroxyl groups on the chitosan-coated surface. The medium intensity peak at 1404 cm^−1^ corresponding to O–H bending confirmed the –OH functional group existence. The weak intensity band at 2873 cm^−1^ corresponded to C–H stretch [36]. Peaks at 1645, 1545, 1373 and 1311 cm^−1^ were assigned to -C = O of amide, -NH_2_, -NHCO of amide and –C-N respectively [37], [38]. The peaks at 1147, 1,070, 1,024 and 897 cm^−1^ were ascribed to the saccharide structure [39]. All these characteristic bands were retrieved from chitosan and the coated surface spectra, proving that the titanium surface was covered by chitosan.

To F-SIMS analysis was performed to confirm the presence of chitosan on the titanium substrate and to control the uniformity of the coatings. The analysis of chitosan coating allowed us to analyze the relative proportions of each detached fragment. First, six specific fragments of chitosan were identified through the analysis of the chitosan solution. These six fragments were also found on the surface of the sample. Table 1 presents the average percentage of these fragments weighed against the total count in chitosan solution and on the coated surface. The proportion of each fragment on the coated surface was of the same order of magnitude as the fragment proportions in the chitosan solution, proving the chitosan presence on the substrate surface. Moreover, the good repeatability between the three different locations shown by the low standard deviations confirms the homogeneity of coating.

### 2. Mechanical properties

Surface chemical analysis could not provide enough information about the adherence of chitosan to the surface. As previous studies have shown that the covalent bond formed between titanium and chitosan via silane induced a significantly higher adherence than a simple chitosan deposition, coating adherence and mechanical resistance should be demonstrated [17], [40]. This mechanical property is a key parameter for implantology.

#### 2.1. Mean stress determination

Vickers Hardness and scratch test were applied to both the titanium substrates and coated samples. The five values of Vickers hardness for each applied load are presented in Figure 3A. For a bulk material and a high indentation force, the mean contact pressure or the hardness (H ∝ HV∝ F/l^2^) is supposed to be a constant of the material, but in the range of microhardness (F<5N) is not true. The decrease of the titanium hardness versus the applied load in the range of microhardness is interpreted by the indentation size effect and the hardness, HV, and may be expressed as [41], [42]:


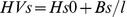
(3)

whereas Hs0 is the hardness of titanium at ‘infinite’ load (taken as 30 kg) and Bs a constant. The same intrinsic microhardness expression may be applied to the coating:


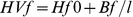
(4)

HVc measurements of the composite (substrate plus coating) result from the influence of both materials and the coating thickness: HVc  =  f (HVs, HVf, e). A scheme of the different curves is presented Fig. 3B. It represents the theoretical curves of the Vickers hardness versus inverse of the diagonal of the indent. The curve of a homogenous bulk material follows the equations (4) and (5). HVs represents the curve of a hard substrate and HVf the curve of a soft film. HVc is the curve of the composite film-substrate: for high load (l → ∞), HVc ∼ HVs, for very low load (l → 0), HVc ∼ HVf. For intermediate loads, the hardness, HVc, varies between these two curves in function of the depth penetration of the indenter and the thickness of the film.

From the results expressed in Fig. 3A it was not possible to precisely determine the hardness of the coating; however, it is necessarily inferior to the composite hardness HVc. Also, hardness values showed that we were in the context of a soft coating (HVf0 <34 HV) on a hard substrate (HVs0  = 150 HV). The stress H applied to titanium can be calculated. In further assays, loads were applied between 5 and 8 N. Mean stress was then calculated for this range of loads as described (part 4 of Materials and Methods): H = 2436 MPa.

#### 2.2. Identification of a damage scenario

Optical micrographs of the scratches are presented in Fig. 4 and Fig. 5. No important delamination was detected but the image of the scratch was made up of two parts: an internal groove (dark part) surrounded by two dark lines parallel to the groove. The theoretical groove diameter, ds-th, induced by the diamond tip was calculated and compared to measurements of the groove diameters on titanium, ds-exp and groove diameters on the coated samples, dc-exp. Two hypotheses were made for an applied load superior at 5 N:

H1: the deformation (the diameter of the groove) is the same in the substrate without coating and the coated sample, ds ∼ dc. In fact, the coating is very soft compared to the substrate, HVs > HVc, and the film thickness, e, is very small compared to the radius of the contact, dc/2, so it fully transmits the stress to the substrate [43], [44].

H2: the scratch test was done in a perfectly plastic regime of the substrate and the sliding mean contact pressure, Hn, is not very different from the mean indentation pressure, H [45].

Taking in account these two hypotheses, theoretical groove diameters on titanium (ds-th) for each load were calculated thanks to the previously evaluated mean contact pressure, H ∼ 2436 MPa.


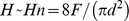
(5)

Hence


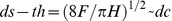
(6)

Theoretical and experimental diameters are represented in Fig. 6. The hypotheses were validated as the titanium experimental points correlated with the theoretical pattern. The groove diameters on the chitosan-coated samples matched the diameters of grooves on the titanium. Also, the dark lines observed on the edges of the groove are due to the bulges induced by plastic deformation of the material.

The hardness determination showed that the coating was not as hard as the titanium. Therefore the most probable damage scenario was that the coating material is pushed to the sides by the tip, thus forming bulges around the groove. Also, the chitosan-coating still adhered to the titanium substrate; the applied stress did not induce peeling off but only a progressive plastic deformation. However, the images of scratches showed a regime change from 5.5 N; the deformation becomes increasingly chaotic, thus inducing high damages to the coating. It is therefore necessary to determine a critical scratching load.

#### 2.3 Determination of a critical load

Our results demonstrated that the coating material was pushed by the tip as it moved. The following assumption can be made: when the tip did not reach the titanium substrate, some coating material seeped under the tip and not on the sides. But when the tip was too close to the titanium, the flow of material could no longer seep under the tip but only on the sides (Fig. 7). This might explain the enlarged bulges and their irregular shape when the applied load was increased. Thus, coating deformation was related to contact depth of the tip, which can be calculated by the following equation:


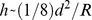
(7)

Where R is the diamond tip radius, d is the groove diameter and d is the groove diameter in millimeters.

According to the profilometry measurements, the chitosan film thickness was e  =  3.83±0.21 µm. The contact depth values of the tip given in Table 2 showed that the tip exceeded the coating thickness from a 5.5 N load; preliminary optical microscopy observations were then confirmed. Dental implants can be subjected to friction force, applied by a toothbrush for example. Indeed, during the healing period, the implant should be brushed on a regular basis, which can cause damages to the coating. That is why we controlled the coating adherence and its scratch resistance. The hardness evaluation and scratch test proved that the coating was strongly adhesive when subjected to scratch. The coating did undergo a deformation but did not have cracks or scales. However, material deformation reached a critical point from a 5.5 N load. This critical load tends to be compared to the distortion caused by a toothbrush or solicitations during the implant placement [46]. To our knowledge, there is no other available data on scratch resistance of this type of covalent coating. The results proved that this type of chitosan coating provides sufficient adherence properties for dental/orthopaedic implant use.

### 3. Biological properties evaluation

#### 3.1 Antibacterial Assessment

The antibacterial activity of the coating was tested and compared to the uncoated titanium substrate activity (Fig. 8). It is known that titanium has no antibacterial activity, it was thus, used as control. The action of the coating was different on the two bacterial strains. Inhibition of bacteria compared to control was observed at 32 h of *P. gingivalis* growth but this result was not statistically significant. In contrast, a strong inhibition of bacterial strain was observed on *A. naeslundii*. For 24 h, no significant bacterial proliferation was observed with coated or with uncoated samples. We can assume that the bacterial growth was affected by the change of environment. After 28 h, a high bacterial growth was noticed with uncoated sample. On the contrary, no bacterial proliferation was detected in contact with chitosan coating. After 28 h of growth, about 60% of the bacterial population was inhibited in the presence of substrate coated with chitosan. This inhibition was statistically significant (p<0,01).

*P. gingivalis* and *A. naeslundii* were chosen among the large diversity of oral microflora because these two bacterial strains are frequently retrieved from peri-implant infection [47], [48]. *A. naeslundii* is a anaerobic Gram positive bacteria which is a major component of dental plaque and among the earliest colonizers of dental surfaces [49], [50]. Previous studies show that *Actinomyces* is a prevalent genus isolated from failed implants and plays an important role at the early stages of tooth surface colonization [51]. Thus the study of our implant device activity on *A. naeslundii* was particularly interesting. Chitosan activity against A. aeslundii had already been demonstrated but with chitosan in solution [52].

It was also interesting to test the effect of coating on a Gram-negative bacterial strain. The obvious choice was to study Porphyromonas gingivalis. *P.gingivalis* is actually one of the bacteria most frequently involved in periodontal diseases and particularly in peri-implantitis [53]–[55]. *P. gingivalis* underwent a lower inhibition than *A. naeslundii* which was not statistically significant. Thus, the coated implant induced two different behaviors in the two bacterial strains. This result was expected because chitosan is known to be more active against Gram-positive bacteria than Gram negative [56].

The results showed that chitosan seemed to maintain its bacteria-repellent behavior after being coated on the titanium. These results are very promising and it would be interesting to test the coating on other bacteria. If chitosan retains its bioactivity when coated, we can assume that this type of material possesses a broad spectrum of activity. Previous studies have demonstrated that chitosan is active against such bacteria as Escherichia coli, Streptococcus mutans, Staphylococcus aureus, Staphylococcus epidermidis or Bacillus subtilis [56], [57]. Thus this type of coating could be bioactive in a large range of applications including dental/orthopaedic implants and medical devices.

#### 3.2. Biocompatibility

#### 3.2.1. Cell proliferation

The results presented in Fig. 9 showed that the percentage of resazurin reduction was practically the same on uncoated titanium substrate and chitosan-coated samples. No statistical differences were noticed between fibroblast proliferation whatever the number of days of growth. This indicated that fibroblast proliferation was not inhibited by the antimicrobial coating. A basic requirement for the use of chitosan-coated implants is that they are biocompatible with respect to fibroblast cells. These results showed that cells proliferate as well on chitosan-coated samples as on titanium. Since titanium materials are known to have a high biocompatibility with bone and soft tissue cells, we can assume that chitosan-coated titanium could also allow cell proliferation on an implant surface [7], [58]. Therefore, such coated implants should be suggested for clinical use.

#### 3.2.2. Cell morphology

The ability of the cells to spread and proliferate on the different substrates was shown by cytoskeletal and nucleus staining and then by confocal microscopic analysis (Fig. 10). The images showed that the fibroblasts are well spread and attached after 2 days of cell culture to each of the surfaces. No round-shaped cells were observed. The fibroblasts had a similar form on the chitosan coating (Fig. 10A) and titanium (Fig. 10B). Cells on chitosan coating formed lamellipodia and had a length of approximately 50 micrometers. Actin filaments were mostly noticeable on the cell edges but seemed to be well organized. The cell morphology confirmed that the chitosan-coated surface was able to support fibroblast attachment and viability as was demonstrated by a resazurin test. The cell density was still low after two days of growth but some cells had begun to spread. Cell proliferation could be observed after 4 and 7 days of growth (Fig. 10C and 10D). The number of fibroblasts increased significantly and cells proliferated according to the material structure (scratches, polish marks). These results were in agreement with previous studies reporting that chitosan has a critical role in cell attachment and growth [59], [60]. Confocal microscopy observations confirmed the viability test and then proved that chitosan-coated material is as biocompatible as chitosan.

## Conclusion

Chitosan was successfully covalently coated to the titanium substrate via TESBA silanation in a two step reaction. To evaluate the efficiency of this type of coating, its chemical, mechanical and biological properties were tested. FTIR and Tof-SIMS analyses confirmed that TESBA was covalently bonded to the surface after silanation and that the finalized sample was homogeneously covered by chitosan. The coating is strongly adhesive to the substrate and has excellent scratch resistance with a critical load of 5.5N. The coated chitosan seemed to maintain its antibacterial properties by inducing 60% inhibition of Actinomyces Naeslundii growth in only a few hours. Moreover, the coated material proved to be non-cytotoxic for NIH3T3 fibroblasts and as biocompatible as titanium. Thus this method of covalent coating provides a biocompatible material with improved bioactive properties. Chitosan-coated implant material therefore has a significant potential in dental implantology and more generally in biomedical device implantation.
